# Self-reported and experimentally induced self-disgust is heightened in Parkinson’s disease: Contribution of behavioural symptoms

**DOI:** 10.1371/journal.pone.0223663

**Published:** 2019-10-16

**Authors:** Marianna Tsatali, Paul G. Overton, Ana B. Vivas

**Affiliations:** 1 South East European Research Center, SEERC, Thessaloniki, Greece; 2 Psychology Department, University of Sheffield, Sheffield, United Kingdom; 3 Psychology Department, The University of Sheffield International Faculty, City College, Thessaloniki, Greece; University of Catania, ITALY

## Abstract

Parkinson’s disease (PD) is associated with deficits in the recognition and expression of basic emotions, although self-reported levels of the self-conscious emotions shame and embarrassment are higher. However, one self-conscious emotion—self-disgust–which has been shown to have a negative impact on psychological wellbeing, has not been investigated in PD before. Here we employed self-report measures of self-conscious emotions, and an emotion induction paradigm involving images of the self, and narrated personal vignettes of instances when patients with PD (and controls) found themselves disgusting. We found that self-reported and induced levels of self-disgust were higher in PD patients than in matched controls, and that trait self-disgust was specifically related to disorders of impulse control in PD patients. Given the link between self-disgust and impaired psychological wellbeing, and the prevalence of anxiety and depression in PD, self-disgust might make a useful therapeutic target for psychological interventions in the condition.

## Introduction

Parkinson’s disease (PD) is a neurodegenerative condition involving the dopaminergic neurons of the substantia nigra, classically characterised by motor symptoms [1–2]. However, a number of non-motor symptoms, such as depression, anxiety, sleep problems, and Impulse Control Disorders (ICDs), are now also recognised to be part of the condition [3]. In addition, over the last two decades there has been growing interest in emotional processes in PD, as a number of studies have reported emotional disturbances in the disorder [4–6]. Emotional disturbances in PD may not be too surprising given the looped architecture of the basal ganglia within which the substantia nigra pars compacta operates, and the clear identification of a limbic (emotional) loop within that architecture [7].

In relation to the basic emotions, changes in PD have been identified in the recognition of emotional stimuli. There is consistency across studies that PD patients are less able to decode emotional features delivered through prosodic elements of the human voice [8–10]. Parkinson’s patients are also impaired at recognising emotions in facial expressions [8–9, 11–14]. The deficits in emotion recognition, both in terms of prosody and facial expressions, appear to be worse for negative emotions [8–9, 15], and importantly are unrelated to depression [9, 4] and medication status [4].

In addition to changes in the recognition of emotional stimuli, changes have also been reported in vocal and facial expressions of emotion. Parkinson’s patients exhibit a reduced pitch and loudness range (‘dysprosody’) when producing emotional speech [16–19], and are impaired when it comes to making spontaneous and posed facial expressions [17, 20–23]. Again, changes in emotional expression, at least in terms of the face, are independent of depression [21,23] and seem to be worse for negative emotions [21–23]. The impairments in vocal expression on the other hand may be broader, involving both sad and happy prosody [19].

In contrast to the widely reported impairments in emotion recognition and emotional expression in PD, experience of the basic emotions seems to be relatively unimpaired, whether that be the subjectively reported valence of emotions induced by images [24–25], or subjective emotional reactions (including amusement) induced by video clips [23,26], although arousal levels appear to be lower in PD patients [24–25,27]. However, the picture is somewhat different when it comes to *self-conscious emotions* rather than basic emotions. Self-conscious emotions are emotions that relate to our sense of self and our awareness of others’ reactions to us [28]. Although these emotions have been considered far less in relation to PD than the basic emotions, and have not been examined before in explicit emotion induction paradigms (unlike the basic emotions–see above), PD patients report increased levels of shame [29–30] and embarrassment [31–35], related to speech and language difficulties and the visibility of their condition. However, one potentially significant self-conscious emotion that has thus far received no attention in PD is self-disgust. Self-disgust is an aversive self-conscious affective state, independent of other self-conscious emotions like shame [36], that reflects disgust directed towards the self [37] and encompasses two dimensions: disgust directed towards one’s physical characteristics (physical self-disgust), and disgust directed towards one’s actions (behavioural self-disgust). Self-disgust has been operationalised as an “emotion schema”, which involves a lasting appraisal of aspect(s) of the self as disgusting [36], and growing evidence suggests that self-disgust is a causative factor in poor psychological wellbeing beyond shame and guilt [36,38].

In relation to PD, self-disgust may be particularly pertinent. Firstly, research has shown elevated levels of self-disgust (linked to poor psychological wellbeing) in other chronic illnesses, both physical [39] and psychological [40–42]. Secondly, we have argued that the self-disgust schema is constructed at least in part from the reading and decoding of emotional reactions in others to enduring characteristics of one’s self and one’s behaviour [36]. As consequence, given that PD is a disorder with a highly visible behavioural syndrome of the classical motor symptoms [1], drug-induced dyskinesias [43], emotional expressivity problems [16–17], and non-motor symptoms like ICDs [44]—all of which break sociocultural rules for ‘normality’–reactions by others to the disorder are likely to be prominent and frequently encountered. We suggest that these reactions are likely to be intense enough to break through any emotional recognition difficulties the patients may have. Indeed, the increased levels of shame and embarrassment in PD [29,31] suggest that patients are very sensitive to their symptoms.

With this in mind, in the present study we have assessed self-reported levels of self-disgust in a group of PD patients (relative to matched controls), and examined a range of potential predictors of self-disgust in this population. Included amongst these are impulsivity, ICDs and the dopamine replacement therapies which appear to play a role in their induction [44], all of which contribute to behaviours which could be causative in establishing a self-disgust schema. In addition, we have evaluated the possibility of inducing self-disgust in PD patients in an experimental context.

We hypothesize that baseline levels of self-disgust will be higher in patients and, given the evidence above that shame and embarrassment can be induced in PD patients (by the visibility of their symptoms to others), we expect it will be possible to similarly induce self-disgust in PD patients and that the extent of that induction will exceed that in controls. Assessing self-disgust and factors that predict it in this population could be the first step in tacking an important causative element in the well-documented poor psychological wellbeing in PD [45]. Also, given the well understood pathological process in PD, alterations in self-disgust might give clues to the neural substrate for the emotion, which as yet remains elusive, except for evidence of amygdala involvement, although in women with borderline personality disorder [46].

## Materials and methods

### Participants

Forty-five patients diagnosed with PD and 45 healthy participants matched on age (in years), gender and education (in years) took part in the study (see Table 1). Patients were recruited from the Outpatients Clinic of the Neurology Department of Papageorgiou and AXEPA Hospitals in Thessaloniki, Greece and the Parkinson Care Association ‘EPICOUROS’ in Athens, Greece. Healthy control participants were recruited from Senior Day Care centers in Thessaloniki and from a research volunteers’ data base from previous studies.

**Table 1:** Demographic and clinical characteristics of the patients with Parkinson disease (PD) and healthy controls at the time of assessment.

*Variables*	PD patients		Healthy controls			
	Mean	S.D.	Mean	S.D.	t	p
Age (years)	71.98	8.60	71.96	9.97	.011	.991
Men % (n)	42% (19)		44% (20)		--	--
Women % (n)	57% (26)		55% (25)		--	--
Education (years)	9.65	4.55	9.28	4.55	.391	.696
Onset disease (years)	9.00	9.14	--		--	--
MMSE	26.76	1.75	27.51	1.85	1.99	.051
Hoehn-Yahr stage	2.13	0.86	--		--	--
HADS-A	7.95	4.79	5.62	3.73	2.515	.014
HADS-D	8.75	4.97	5.06	3.38	4.530	<.0001
LEDD	511.20	251.23	--		--	--
QUIP-RS	8.37	10.24	--		--	--

MMSE = Mini-Mental State Examination; HADS-D = Depression subscale from the Hospital Anxiety and Depression Scale; HADS-A = Anxiety subscale; LEDD = Levodopa equivalent daily dose (mg); QUIP-RS = Questionnaire for Impulsive-Compulsive Disorders in Parkinson’s—Disease Rating Scale

The inclusion criteria for the patients were: i) diagnosis by a neurologist according to the UK Parkinson’s Disease Society Brain Bank Clinical Diagnostic Criteria [47], ii) to be at the mild or moderate stage of the disease according to *The Unified Parkinson’s Disease Rating Scale–*UPDRS- [48–49] or/and the *Hoehn &Yahr scale* [50], iii) a score on the Mini-Mental State Examination–MMSE- [51] equal to or above 24 [52], iv) no evidence of other types of neurological or psychiatric diseases (except for anxiety and depression), such as atypical parkinsonism, idiopathic tremor, parkinsonism syndromes or vascular parkinsonism; and v) no evidence of a history of alcohol and other drugs abuse. The inclusion criteria for the control group were: i) a score on the MMSE equal to or above 24; ii) no history of self-reported neurological or psychiatric disease, of alcohol and/or other drug abuse.

The study, including the validation of the SDS-Greek (the see S1 Appendix. Validation study of the SDS-Greek), was approved by the University of Sheffield Ethics Committee, and informed written consent was obtained from all the participants. Patients included in the study were selected together with the clinician on the basis of being able to understand the requirements of the study. Thus, they were judged competent to provide informed consent. Almost all patients (42) were under medication (combinations of levodopa, inhibitors of dopamine catabolism and dopamine receptor agonists) at the time of the study. The levodopa equivalent daily dose (LEDD) was calculated using the formula proposed by [53].

### Measurements and procedure

#### Self-report measures

The Hospital Anxiety and Depression Scale–HADS- [54–55] was employed to measure depressive and anxious symptomatology, which has been consistently correlated with self-disgust scores [37–38]. The HADS includes 14 items, with a scale ranging from 0 (not at all) to 3 (most of the time). The maximum score in the anxiety and depression sub-scales is 21.

The Questionnaire for Impulsive-Compulsive Disorders in Parkinson’s—Disease Rating Scale -QUIP-RS- [44] uses a 5-point scale, from 0 to 4, to measure frequency of compulsive thoughts and impulsive behaviours in connection with the targeted category (gambling, sex, buying, hobbyism and punding). The total combined score for QUIP-RS ranges from 0 to 112. The scale was translated and back-translated by two bilingual Greek-English neuropsychologists for the purpose of this study.

The Barratt Impulsiveness Scale -BIS-11- [56–57] was employed to measure behavioral and trait of impulsiveness. The tool consists of 30 items, and the participant is required to answer each item on a 4-point scale from 1(Rarely/Never) to 4 (Almost Always/Always). The overall scale consists of 3 subscales; Motor Impulsiveness, Attentional Impulsiveness, and Non-planning Impulsiveness.

The Self-Conscious Affect questionnaire–TOSCA- [58–59] was employed to measure shame and guilt. The scale consists of 16 scenarios, and participants self-report their potential reactions using a 5-point scale. TOSCA-3 includes six subscales, shame-proneness, guilt-proneness, externalization, detachment/unconcern, A-pride and B-pride. In this study we analysed only the shame and guilt subscales.

The Self-Disgust Scale (SDS) was developed by [37] to measure disgust directed towards the Self. This scale has not been used before in the Greek population, so we developed a Greek version of the scale (SDS-Greek, or SDS-G) and conducted a validation study (see S1 Appendix. Validation study of the SDS-Greek).

#### Emotion induction experiments

To our knowledge, there are no previous studies concerning the experimental induction of self-disgust, since self-disgust is a relatively new construct. Thus, we designed two experiments to induce disgust (and other emotions) toward the self.

Narration-induction experiment. This experiment was based on the paradigm to induce self-conscious emotions developed by Dickerson and colleagues [60], in which participants were asked to write down an experience that made them feel a certain way. Participants sat comfortably in a chair, and were instructed to narrate orally instead of writing (on the assumption that the PD patients would find oral narration less taxing) experiences that made them feel ashamed, guilty or self-disgusted. Also they were asked to narrate a neutral experience, something they did the day before. There was no time limit and narrations were recorded using Audacity software.

The guidelines for the narrations’ induction paradigm were similar to those of Dickerson et al. [60]. However, in Dickerson et al. [60] participants were asked to write about one traumatic and upsetting experience that made the individual feel bad about him/her-self or blame his/her-self, whereas in our study, participants narrated three separate traumatic and upsetting experiences in which they felt self-disgust, shame and guilt. Specifically the instructions for self-disgust were as follow ‘*I want you to narrate one of the most traumatic and upsetting experiences of your life; please focus on an experience that you felt disgust towards the self*. *It could be an experience which made you feel negatively about yourself or a past experience when you did not like yourself*. *The important thing is that you tell about your deepest thoughts and feelings*. *Ideally*, *whatever you speak about should deal with an event or experience that you have not talked with others about in detail*’. The same instructions were given for guilt and shame but changing the wording so that participants were encouraged to narrate an incident which made them feel guilty and ashamed, respectively. The instructions for the neutral condition were again similar to those employed in Dickerson et al. [60], ‘*I want you to tell about what you did during the past 24 hours*. *You should describe your activities and schedule in detail*, *discussing the facts and circumstances as objectively as possible*. *You might describe what you had for dinner last night*, *what time you got up this morning*, *and so forth*. *The important thing is you discuss the facts and try to remain objective about your activities*’.

After each narration, participants were asked to self-report how they felt using a Visual Analogue Scale (VAS) from 0 (Not at all) to 100 (Extremely). Specifically, they were asked to self-rate the target emotion in the narration (self-disgust, shame or guilt), other non-target emotions (anger, sadness and happiness), and level of arousal (from 0 -I feel completely calm- to 100 -I feel completely excited-).

Photo-induction of self-disgust. Participants were presented either with a full body picture of themselves or with a neutral picture. On arrival at the experimental setting, and after obtaining informed consent, the participants were asked to sit and pose with a neutral posture while the researcher took a photograph. In the paradigm, this was contrasted with a neutral photo selected from the International Affective Picture System, which had neutral scores in pleasantness and arousal, depicting hanging clothes (n.7217). Each photo was presented for 3 s, and the order of the photos was counterbalanced across participants. Participants were asked to view the photos passively, and were instructed as follows, “*You will be presented with two consecutive photos*. *Please look at each photo carefully*. *After each photo you will be asked to rate how you feel”*.

After each photo, participants were asked to self-report the target emotion (self-disgust), other non-target emotions (anger and sadness), and arousal levels using Visual Analogue Scales (VASs).

#### Procedure

Data collection lasted three hours approximately, and took place into two sessions. The first session included demographic data and the medical history of the participant, and all the self-report measures. The second session consisted of the emotion induction experiments. Testing took place in a quiet room in the health centers, the Senior Day Care centers, or at the University of Sheffield International Faculty. The picture-induction and the narration-induction procedures took place in a counterbalanced order across participants, and with a break in between.

### Statistical analysis

Statistical analyses were carried out using IBM SPSS Statistics (Version 24). Comparisons between participants with and without PD on self-report measures and VAS measures of self-conscious emotions were carried out using Multivariate Analyses of Variance/Analyses of Variance. We further include as co-variate in the analyses those key confound variable that differed significantly between the groups. Pearson’s correlations were undertaken to investigate the relationship between demographic, clinical, psychological predictor variables and self-conscious emotion outcome variables. Regression analyses were conducted to investigate the relationship between predictor variables and the SDS-G outcome measure. Given the high number of correlations between the variables, only those that were significant at p < .01 were entered into the regression analyses [61]. The assumptions for linear regression analyses were examined and found satisfactory. In particular, standardized residual scatterplots followed the normal distribution with no obvious outliers. Therefore, analyses proceeded as planned.

## Results and discussion

Five patients declined to participate in the emotion induction experiments. Consequently, 40 PD patients and 40 matched HC were included in the VAS analyses. From these 80 participants; five (3 HC and 2 PD) could not recall an experience were they felt disgust towards the self, eight (4 HC and 4 PD) could not recall an experience were they felt ashamed, and three patients could not recall an experience were they felt guilty. Thus, data from 80, 75, 72 and 77 participants were included in the Photo, Self-disgust narration, Shame narration and Guilt narration analyses, respectively. As shown in Table 1, PD patients and HC did not differ significantly on any of the demographic variables (age, gender and education). However, PD patients had significantly higher scores in anxiety and depression than HC participants.

### Analysis of self-reported self-conscious emotions

The mean scores for the self-conscious emotion self-report questionnaires were submitted to a one-way MANOVA with group (PD and healthy control, HC) as the between subject factor and scores from the SDS-G, and the subscales of Shame and Guilt (TOSCA) as dependent variables. According to Pillai’s Trace there was a significant main effect of group on self-conscious emotions, [V = .164, F(3, 85) = 5.568, p = .002, ηp^2^ = .164]. Univariate analyses showed that the PD (Mean_SDS_ = 31.36 and Mean_Shame_ = 47.55) group had significantly higher scores than the group of HC (Mean_SDS_ = 23.04 and Mean_Shame_ = 42.69) on Self-disgust and Shame, F(1, 87) = 5.568,p = .001,ηp^2^ = .117] and [F(1, 87) = 4.079, p = .047, ηp^2^ = .645], respectively. There were no significant differences between the groups (Mean_PD_ = 64.52 and Mean_HC_ = 64.15) on Guilt scores, [F(1, 87) = .039, p = .843, ηp^2^<.0001].

Since PD patients and HC significantly differed on depression and anxiety scores, and depressive symptoms scores significantly correlated with Self-disgust and Shame scores, and anxiety symptoms with Shame scores, we conducted a further MANCOVA with Group as the between subject factor and depression (HADS-D) scores as a co-variate. According to Pillai’s Trace, the main effect of group on self-conscious emotion did not reach statistical significance, [V = .073, F(3, 84) = 2.198, p = .094]. However, the univariate analyses showed that the main effect of group on Self-disgust scores continued to be significant after including depression symptoms as a co-variate, [F(1, 86) = 4.457, p = .038, ηp^2^ = .049]. A further MACOVA with anxiety scores as a co-variate (HADS-A), showed a significant main effect of diagnosis on self-conscious emotions according to Pillai’s trace [V = .118, F(3, 84) = 3.74, p = .014, ηp^2^ = .118]. Univariate analyses showed that the main effect of group on Shame scores was no longer significant, although the main effect of Group on Self-disgust scores remained significant [F(1, 86) = 7.38, p = .008, ηp^2^ = .079].

### VAS analyses for the Photo-induction experiment

The MANOVA (see Table 2) with the VAS scores from the Photo emotion-induction experiment showed a significant main effect of Condition (self-photo vs neutral photo), according to Pillai’s Trace [V = .072, F(4, 75) = 47.61, p <.0001], and a significant Group by Condition interaction, [V = .131, F(4, 75) = 2.82, p = .031]. Further, 2x2 ANOVAs on each VAS emotion showed that for self-disgust, the main effects of Group and Condition, and their interaction were statistically significant, [F(1, 78) = 4.35, p = .040, ηp^2^ = .053], [F(1, 74) = 74.95, p<.0001, ηp^2^ = .490], and [F(1, 78) = 3.98, p = .050, ηp^2^ = 0.049], respectively. PD participants had significantly higher self-disgust VAS scores than HC participants but only in the self-photo condition. For the anger VAS scores, results showed significant main effects of Group, Condition, and their interaction, [F(1, 79) = 5.96, p = .017,ηp^2^ = .071],[F(1, 78) = 11.65, p = .001, ηp^2^ = .130], and [F(1, 78) = 8.79, p = .004,ηp^2^ = .101], respectively. The analysis of the interaction showed that PD participants had significantly higher anger VAS scores than the HC participants but only in the self-photo condition. For the sadness VAS scores, results showed significant main effects of Group and Condition, F(1, 78) = 5.10, p = .027, ηp^2^ = .061] and [F(1, 79) = 48.24, p < .0001,ηp^2^ = .382] respectively. That is, PD patients had overall higher sadness VAS scores than HC participants, and scores were also higher for the self-photo condition than for the neutral photo condition. Finally, for the Happiness VAS scores, results showed only a significant main effect of Condition [F(1, 79) = 30.53, p <.0001, ηp^2^ = .281].

**Table 2:** Mean VAS scores and SD as a function of Group and Condition for the photo emotion induction experiment.

		PD		HC			
VAS emotion	Condition	Mean	S.D.	Mean	S.D.	*t*	*p*
Self-Disgust	Self	38.63	34.25	24.00	29.68	2.04	.045
	Neutral	0.25	1.58	.00	.00	1.00	.320
Anger	Self	15.00	27.36	2.25	9.99	2.77	.007
	Neutral	.75	3.49	1.25	6.48	.429	.669
Sadness	Self	32.50	32.09	18.75	28.66	2.02	.047
	Neutral	5.50	17.53	.75	4.74	1.65	.102
Happiness	Self	29.50	32.02	38.37	38.70	1.12	.267
	Neutral	14.25	25.51	7.75	21.06	1.24	.218

### VAS analyses of the Narration-induction experiment

The 3 separate MANOVAs with VAS scores from the narration emotion induction experiment showed the following results (see Table 3). For the self-disgust narration induction, there were significant main effects of Group and Condition (self-disgust narration vs neutral narration) and their interaction, [V = .180, F(4, 69) = 3.77, p = .008], [V = .862, F(4, 69) = 108.14, p <.0001] and [V = .172, F(4, 69) = 3.59, p = .010], respectively. We then conducted 4 separate 2x2 ANOVAs for each VAS emotion. For the self-disgust VAS scores, results showed significant main effects of Group, Condition, and their interaction, [F(1, 72) = 13.38, p <.0001, ηp^2^ = .157], [F(1, 72) = 314.36, p <.0001, ηp^2^ = .814] and [F(1, 72) = 13.66, p <.0001, ηp^2^ = .160], respectively. The analysis of the interaction showed that PD patients had higher self-disgust VAS scores than HC participants only in the self-disgust narration condition. For the anger VAS scores, results showed significant main effects of Group and Condition, [F(1, 72) = 4.86, p = .031, ηp^2^ = .063] and [F(1, 72) = 93.79, p <.0001, ηp^2^ = .566], respectively. That is, PD participants had overall higher anger VAS scores than HC participants, and the anger VAS scores were also higher in the self-disgust narration relative to the neutral narration. For the sadness VAS scores, results showed significant main effects of Group, Condition and their interaction, [F(1, 72) = 5.90, p = .018, ηp^2^ = .076], [F(1, 72) = 119.63, p <.0001, ηp^2^ = .624] and [F(1, 72) = 4.97, p = .029, ηp^2^ = .065], respectively. The analysis of the interaction showed that PD participants had significantly higher sadness VAS scores than HC participants only in the self-disgust narration condition. For the happiness VAS scores, results showed a significant main effect of condition.

**Table 3:** Mean VAS scores and SD as a function of Group and Condition for the Self-disgust narration induction.

	Self-Disgust Narration			Shame Narration			Guilt Narration			Neutral Narration		
VAS	PD	HC	*t*	PD	HC	*t*	PD	HC	*t*	PD	HC	*t*
SD	66.84 (27)	44.19 (25)	3.68**	---	---	---	---	---	---	2.70 (11)	1.35 (8)	.62
Shame	---	---		64.72 (27)	49.31 (28)	2.38*	---	---		.00 (.00)	.00 (.00)	---
Guilt	---	---		---	---		70.81 (29)	55.62 (25)	2.45*	.27 (2)	.00 (.00)	1.01
Anger	55.00 (40)	38.78 (36)	1.82	45.22 (36)	39.86 (36)	.63	58.11 (37)	45.75 (35)	1.50	5.41 (21)	.81 (5)	1.33
Sadness	62.63 (33)	43.38 (35)	2.44*	54.17 (34)	47.08 (35)	.87	57.51 (34)	57.13 (33)	.05	5.41 (18)	4.59 (17)	.29
Happiness	3.68 (11)	2.70 (11)	.37	4.17 (13)	.56 (3)	1.65	1.08 (5)	1.25 (8)	.11	48.65 (38)	42.16 (36)	.56

*p<.05;

**p<.001

For the shame narration induction, there was a significant main effect of Condition [V = .846, F(4, 67) = 92.06, p < .001]. That is, overall VAS scores were higher for the shame narration induction condition relative to the neutral narration condition for all the VAS scores: shame, [F(1, 70) = 309.66, p <.0001, ηp^2^ = .816], anger [F(1, 70) = 79.85, p <.0001, ηp^2^ = .533], sadness [F(1, 70) = 101.48, p <.0001, ηp^2^ = .533], and happiness, [F(1, 70) = 87.83, p <.0001, ηp^2^ = .556]. However, only for shame VAS scores, the Group by Condition interaction was also statistically significant, [F(1, 70) = 5.66, p = .020, ηp^2^ = .075]. The analysis of the interaction showed that PD patients had higher shame VAS scores than HC but only in the shame narration condition.

For the guilt narration induction, the MANOVA yielded only a significant main effect of Condition [V = .864, F(4, 72) = 114.79, p <.0001]. Overall, VAS scores were higher for the guilt narration condition relative to the neutral condition: guilt, [F(1, 75) = 417.59, p <.0001, ηp^2^ = .848], anger, [F(1, 75) = 119.11, p <.0001, ηp^2^ = .614], sadness [F(1, 75) = 138.44, p <.0001, ηp^2^ = .649], and happiness [F(1, 75) = 106.17, p <.0001, ηp^2^ = .556]. However, only for the guilt VAS scores, the Group by condition interaction reached also statistical significance, [F(1, 75) = 5.83, p = .018, ηp^2^ = .072]. The analysis of the interaction showed that PD patients had higher guilt VAS scores than HC participants only in the guilt narration condition.

Since only the depression scores significantly correlated with VAS scores both in the photo and the narration emotion induction experiments, we conducted further 2x2 (4) MANCOVAs with depression scores as a co-variate. In the photo-induction, only the main effect of Condition for the Self-disgust VAS scores and the Happiness VAS remained significant after adjusting for the effect of depression, [F(1, 77) = 3.89, p = .05, ηp^2^ = .048] and [F(1, 77) = 12.68, p = .001, ηp^2^ = .141]. In the narration induction, for the Shame narration induction, and the Guilt narration induction analyses, only the main effect of Condition remained statistically significant after controlling for depression scores. However, in the Self-disgust narration induction, the group by condition interaction remained significant for the Self-disgust VAS scores after adjusting for the effect of depression, [F(1, 71) = 5.275, p = .025, ηp^2^ = .069]. That is, PD participants (adjusted Mean = 64.88) had significantly higher Self-disgust VAS scores than HC participants (adjusted Mean = 47.41) in the Self-disgust narration condition; whereas there were no differences between the groups in the neutral narration condition (adjusted Mean_PD_ = 2.88 and adjusted Mean_HC_ = 1.17).

### Regression analyses in the PD group

In the PD group (see Table 4), SDS-G scores had significant positive correlations with LEDD [r = .448, N = 45, p = .002], BIS-Attentional [r = .420, N = 45, p = .004], BIS-Motor [r = .440, N = 45, p = .002] and QUIP-RS scores [r = .624, N = 45, p = .0001], and significant negative correlations with age [r = -.306, p = .041], TOSCA-Guilt [r = -.324, N = 45, p = .007], and TOSCA-Shame [r = -.304, N = 45, p = .045]. TOSCA-Shame scores also had a significant positive correlation with TOSCA-Guilt [r = .567, N = 45, p <.001], and a negative correlation with BIS-Attentional [r = -.305, p = .044], while TOSCA-Guilt was also positively correlated with MMSE scores [r = .401, N = 45, p = .007].

**Table 4:** Correlations between demographic, clinical, psychological predictor variables and self-conscious emotion outcome variables in the group of PD patients.

	1.Age	2.Duration	3.LEDD	4.MMSE	5.HADS-D	6.HADS-A	7.BIS-A	8.BIS-M	9.BIS-NP	10.QUIP	11.TOSCA-S	12.TOSCA-G	13.SDS-G
2.	.188												
3.	-.353*	.168											
4.	-.568**	-.045	.282										
5.	-.188	-.007	-.187	.215									
6.	-.213	.025	.010	.167	.685**								
7.	-.315*	-.172	.330*	.337*	.063	.237							
8.	-.084	-.176	.283	.146	-.121	-.025	.335*						
9.	-.191	-.142	.142	.264	-.128	-.108	.260	.622**					
10.	-.568**	-.096	.582**	.396**	.110	.101	.392**	.417**	.366**				
11.	.025	.018	-.121	.108	.074	-.023	-.305*	-.174	.033	-.113			
12.	-.121	.059	.032	.401**	-.094	-.175	-.139	-.210	-.048	-.062	.567**		
13.	-.306*	-.103	.448**	.104	.105	.230	.420**	.440**	.163	.624**	-.304*	-.324*	

Duration = Years from diagnosis of PD; LEED = l-DOPA equivalent dose; HADS-D = Depression subscale; HADS-A = Anxiety subscale; BIS-A = Attentional subscale of Barratt Impulsiveness Scale; BIS-M = Motor subscale; BIS-NP = Non-planning subscale; TOSCA-S = Shame subscale; TOSCA-G = Guilt subscale;

*p<.05;

**p<.001.

In order to test the hypothesis that symptoms associated with PD may account for (predict) the self-disgust observed in this group, we introduced the significant correlations (p < .01) between SDS-G scores and LEDD, BIS-Attentional, BIS-Motor, and QUIP-RS into a regression model. Scores from the SDS-G were entered as the outcome variable, with higher scores representing higher levels of self-disgust. The regression analysis resulted in a statistically significant model that explained *A*R^2^ = 38% (*R*^*2*^ = .433, *p* <.0001) of the variance in Self-disgust scores, with QUIP-RS being the only statistically significant independent predictor (*β* = .552, *p* = .021).

Impairments in emotion recognition and emotional expression in relation to the basic emotions are widely reported in PD [8,22], although experience of the basic emotions seems to be relatively unimpaired [26]. However, the picture is rather different for self-conscious emotions, where PD patients report increased levels of shame [29] and embarrassment [31]. Here, we sought to investigate for the first time in PD another negative self-conscious emotion–self-disgust–a self-conscious emotion that has been previously demonstrated to play a causative role in depression [62]. In the present study we assessed self-reported levels of self-disgust in a group of medicated PD patients (relative to matched controls), and evaluated the possibility of inducing self-disgust in PD patients in an experimental context. The main findings were that self-disgust levels were higher in PD patients than in controls, even when controlling for the effect of depressive and anxiety symptoms, and that self-disgust levels were significantly and selectively predicted by ICDs as measured by the QUIP-RS. In addition, self-disgust was more readily induced in PD patients than in controls.

Levels of shame were also higher in PD patients, consistent with the existing literature [29–30]. Although, after controlling for depression and anxiety symptoms, levels of shame were no longer found to be higher in the PD group. In the PD group, self-disgust was found to correlate with two aspects of BIS, namely BIS-Attentional Impulsiveness and BIS-Motor Impulsiveness. Increased impulsivity in PD is well recognized. Parkinson’s disease patients exhibit impaired decision making, as well as greater risk taking [63]. In relation to motor impulsivity, PD patients make significantly more impulsive motor errors [64], and have impaired stop-signal inhibition [65]. However, in relation to self-disgust, rather than self-reported trait impulsivity (as measured by BIS), behavioural/clinical symptoms of impulsivity (as measured by the QUIP-RS) were the only significant predictor.

Clinically diagnosed ICDs have a prevalence rate of around 14% in PD [66–67], which rises to 59% when undiagnosed ICDs are also accounted for via the QUIP-RS [68]. Specifically, ICDs are behavioral disturbances characterized by impulsivity, in which patients are unable to control their impulses to act out in ways that compromise social rules [69]. Impulse Control Disorders identified in PD involve a variety of behaviors such as compulsive gambling, shopping, excessive hobbyism, compulsive sexual behavior and binge-eating [68,70]. These disturbances can cause serious problems in patients’ lives [71]. Although, (perhaps not surprisingly given the proposed causal role of dopamine replacement therapies in ICDs in PD [67]) medication (l-DOPA equivalent dose) was correlated with QUIP-RS scores in the present study, its role as an independent predictor of self-disgust was not significant alongside QUIP-RS scores themselves.

Since the predictive relationship between QUIP-RS scores and self-disgust in the present study was based on cross-sectional data, the directionality of the relationship cannot be inferred from the data alone. However, theory would most likely suggest that ICDs (at some level) cause self-disgust rather than the other way round. Self-conscious emotions include the ability to evaluate the self in comparison to others [72], and so they require evaluation of the self and others [28]. Unlike the six basic emotions, self-conscious emotions rely on more complex functions, which are more cognition-dependent. Hence, self-conscious emotions arise in the context of social networks [73], where the sense of the self is depicted in relation to others and with respect to the social norms [74]. As a consequence, being impelled to behave in certain ways by the compulsions that underlie ICDs could be seen as likely to lead to the violation of social norms and thus to provide disgust-related evidence pertaining to the self that gets added to an evolving self-disgust schema [38], leading to heightened levels of trait self-disgust in PD patients. The converse, where self-disgust leads to ICDs, is much more difficult to defend conceptually, especially given that self-disgust tends to lead to social avoidance [38].

In addition to trait self-disgust in PD patients, we also investigated to what extent state self-disgust might be inducible in PD patients (as has been shown for shame and embarrassment–see Introduction)–i.e. how malleable *state* self-disgust was, a question we approached with two experimental emotion induction paradigms. The findings agree with those of self-reported trait levels of SD. That is, PD patients reported higher levels of state self-disgust than healthy controls in experimental conditions where we used an emotional elicitor (self-photo or instruction to narrate an emotional experience), but no differences when the elicitor was neutral. Since we used different scales to measure trait and state self-conscious emotions, we cannot directly compare the results to conclude on the typically reported *intensity or impact bias* (whether trait or state levels are higher) [75]. Previous studies have supported a discrepancy between the mean-level self-reported trait and state scores on emotion, which has been explained in terms of differential cognitive processes and contextual information contributing to the corresponding assessment method [75–76]. Despite the potential differences between our assessment methods, evidence from both methods in the current study converges to increased self-disgust in PD patients.

The use of visual or auditory stimuli such as film and pictures to elicit basic emotions is widespread in the literature [77–79], but to our knowledge our study is the first to use a visual stimulus—a self-photo—to elicit self-disgust. Our study shows that this paradigm, and a paradigm using the reproduction of past emotional experiences (previously used to induce shame in a healthy population) [60] are effective at eliciting self-disgust in PD patients and healthy controls. In both paradigms, participants also reported overall higher levels of non-target negative emotions (anger and sadness) in the experimental condition relative to the neutral condition. This is not an unexpected result, since research has shown that negative emotions tend to co-exist [80], specifically it has been shown that self-disgust and sadness are closely related [62,81]. In addition, the intensity of the state emotion reported using VAS in the self-photo and the self-disgust narration conditions were significantly correlated (r = .349, N = 75, p = .002), which supports the conclusion of both paradigms being effective in eliciting the target emotion.

While the current report represents a useful addition to understanding self-conscious emotions in PD, there are limitations worth noting. The first limitation is that the present research relies entirely on self-report measures. However, self-report measures have been extensively used in research on disgust as they are inexpensive, easy to administer (in comparison to physiological and neurological measures), and are particularly useful in studies (such as this) that are concerned with the simultaneous assessment of multiple emotional states [82]. Furthermore, emotions were measured in two different ways, with questionnaires and VASs, both of which produced conclusions that pointed in the same direction.

The second limitation is that the Greek version of the SDS was validated in a young population rather than older participants (to age match the participants in the main study). This was to allow us to compare the findings to the original English validation. However, scores on the SDS-G (see S1 Appendix. Validation study of the SDS-Greek), did not differ markedly between the validation sample and the older HC group (Mean_Validation_ = 28.0, SD 10.7, and Mean_HC_ = M 23.0, SD 9.1). The small difference agrees with the general finding that self-disgust levels tend to decrease a little with age (see the negative correlation in Table 4).

Another limitation is that although we excluded participants with a potential diagnosis of dementia (based on MMSE cut off scores), we cannot rule out that some of our participants may have fulfilled the criteria for the more recent clinical construct of Mild Cognitive Impairment [83]. Mild Cognitive Impairment is an intermediate stage between healthy aging and dementia, which is predominantly characterized by memory impairments [83], and consequently it might have affected the experiencing and expression of emotions in our participants. However, both PD and HC would have been equally affected, and research suggests that self-reported affect, and memory for emotional scenes are relatively intact in MCI [84–85]. In terms of medical status though, one thing that would have selectively affected the PD group is that the majority of the patients were under medication at the period of testing, and thus we cannot exclude the possibility that the observed results may be due to medication. However, it is important to point out that although LEDD did indeed significantly correlate with self-disgust scores, it was not an independent predictor in the regression model. Thus, the regression model suggests that impulsive behavior was the main factor accounting for the variance in self-disgust scores. Future studies may though seek to replicate the study in patients off medication.

A final limitation is that that the study had a cross-sectional design. However, longitudinal studies are very difficult to conduct and interpret in people with PD, who have a chronic progressive illness. Furthermore, we have found the attrition rate (particularly with negatively-valenced studies like our own) to be high in such groups.

## Conclusions

This is the first study to investigate self-disgust in PD patients. We found that PD patients exhibited higher levels of self-reported, and experimentally induced, self-disgust relative to matched control participants. Additionally, trait self-disgust levels were significantly and selectively predicted by disorders of impulse control in the group of patients. The high levels of self-disgust in PD may represent a useful therapeutic target for psychological interventions. Recently, Acceptance and Commitment Therapy (ACT) has been proposed as a useful approach for psychological distress in chronic illness [86]. Our findings here which stress the importance of emotional factors in PD adds further weight to this suggestion, given ACT’s focus on emotional acceptance. Indeed, ACT has already been found to be effective in treating wearing off anxiety in PD [87].

## Supporting information

S1 Appendix Validation study of the SDS-Greek. (DOCX)
