# Supplementary material for: Self-reported and experimentally induced self-disgust is heightened in Parkinson’s disease: Contribution of behavioural symptoms
Source: PLoS One. 2019 Oct 16;14(10):e0223663. doi: 10.1371/journal.pone.0223663 (PMC6799866; doi:10.1371/journal.pone.0223663)
Supplement: S1 Appendix — (DOCX) [file pone.0223663.s001.docx]

**Validation of the SDS-Greek (SDS-G)**

In total, 250 students, 55 males and 195 females, from Universities in North Greece with a mean age of 22.3 years (range = 18-32, SD = 3.3) participated in the validation study. The mean time spent in education was 14.6 (SD=1.53) years.

The English *Self-Disgust Scale* -SDS- [1] was translated according to Hambleton’s guidelines [2], by two English-Greek bilinguals. The first translation and the back-translation versions were compared for consistency, relevance and meaning of the content. The SDS consists of an 18-item psychometric tool (with 6 filler items), and scores can range from 12 to 84.

To test for construct validity we used the following additional measures. The Disgust Scale – Revised (DS-R) [3], Beck Depression Inventory II (BDI) [4,5], Rosenberg's Self-Esteem Scale (RES) [6,7] and Self-Description Questionnaire (SDQ) [8,9]. All these scales have been validated for the Greek population, and have shown good psychometric properties.

After providing written informed consent, participants completed all the self-report measures. The session lasted for approximately 30 min. Three months after the initial assessment, 16 participants, randomly chosen from the original sample, were administered the SDS a second time for test-retest reliability.

***Reliability and validity***.

Scores on the SDS-Greek (SDS-G) were similar to those of the original test population for the English version in [1] (English – *M* = 29.8, *SD* = 11.9; SDS-G – *M* = 28.0, *SD* = 10.7). The SDS-G had a very high Cronbach’s alpha coefficient for the 12 items, (α=.86). All the items correlated positively and significantly with the total score of the scale (all r > .50, and all p < .0001). The test-rest reliability was satisfactory. That is, there was a significant positive correlation between the total scores of the SDS-G at Time 1 and Time 2, r = .883, N=16, p <.0001.

The concurrent validity of the SDS-G was assessed by testing whether the scores were significantly correlated (see S1A Table) with the DS-R, BDI as in [1], RES as in [10] and the SDQ, on which the English version of the SDS was based [1]. As with the English version, the SDS-G scores were positively correlated with scores on the BDI [r = .651, N=244, p <.0001], and negatively correlated with the scores of the RES [r=-.704, 244, p <.0001], and the SDQ [r=-.507, N=232, p <.0001]. However, the SDS-G was not correlated with the DS-R [r = .013, N=243, p = .841].

-Insert S1A Table about here-

***Factor structure***. In order to test whether the SDS-G maintained the same factor structure as the original English SDS, we conducted Confirmatory Factor Analysis (CFA) using the Structural Equation Modeling program AMOS 20.0 [11]. The original SDS consisted of two specific constructs, *Disgusting Self* (items 1, 4, 6, 10 and 15) and *Disgusting ways* (items 3, 9, 12, 17 and 18). CFA was conducted using maximum likelihood estimation and calculated the covariance matrix among the SDS items [11-12]. We assessed the fit of the model according to the criterion values proposed by [13].

The matrix of loadings between the items and the factors for the SDS-G showed that the 5 items loading on Factor 1 (Disgusting self) in the English SDS had overall higher loadings than those loading on Factor 2 (Disgusting ways). Specifically, 2 of the items loading on Factor 2 in the original SDS, had substantially lower loadings (<.50). Overall, loadings for the two factors in the English SDS were higher than those observed for the SDS-G (see S1B Table).

-Insert S1B Table about here-

The 2 factor model did not show an acceptable fit of the data for the SDS-G (*χ^2^*=132.59, *df*=32, *p<* .001, CFI= 0.877, SRMR= .139, RMSEA= .113). Based on these results, it can be concluded that the SDS-G seems to have a different factor structure to the original English scale. We then conducted CFA to test the single factor model. The single factor model showed an adequate/good fit of the data for the SDS-G (*χ^2^*=52.79, *df*=43, *p* =.146, CFI= 0.973, SRMR= .059, RMSEA= .051).

Our Greek version of SDS (the SDS-G) was found to have high internal reliability, as well as test-retest reliability. Although there are some differences between the original English self-disgust scale (SDS) and the new Greek version (SDS-G), we believe there are sufficient pertinent similarities (and reasons for the dissimilarities) to support the validity of the SDS-G. Scores on the SDS-G - tested on a student population - were similar to those of the original test population for the English version (again, primarily students) in Overton et al. (2008). Furthermore, scores on the SDS-G were correlated with those of RES and the BDI, as are those of the English version. Although there was no correlation of the SDS-G with the Disgust Scale, unlike the small (r = .25) correlation between the Disgust Scale and the English version, our work with the SDS-G used the revised version of the Disgust Scale (DS-R), for which a well validated Greek translation existed, rather than the earlier Disgust Scale used for the English SDS, which does not have such a translation. Scale differences may explain the absence of a small correlation in the case of the SDS-G. The lack of a correlation with the DS-R is not a major difficulty for validity since we conceptualise self-disgust to be largely distinct from other types of disgust [14].

Cultural differences are likely to play a part in the second difference between the SDS-G and the English SDS, namely that the former (unlike the latter) did not decompose to two subscales when factor analysed. Cultural differences may well affect the way self-disgust is experienced [15-16]. For instance, Great Britain and Greece differ on the cultural dimension of individualism/collectivism [17,18], and research has shown that more individualist cultures tend to experience self-conscious emotions as an external concept, whereas more collectivistic cultures tend to evoke self-conscious emotions more frequently and intensely [19]. In addition, moral codes, the violation of which are likely to contribute to self-disgust [14], are liable to have somewhat different underpinnings in a society like that of Greece, with a dominant influence of orthodox religion, than in society like that of the UK, which is both liberal and secular. Regardless, since the SDS-G possess good psychometric properties (internal consistency, test-retest reliability), we conclude that the SDS-G is a useful tool to measure self-disgust in the Greek population as a single factor.

**References**

1. Overton PG, Markland FE, Taggart HS, Bagshaw GL, Simpson J. Self-disgust mediates the relationship between dysfunctional cognitions and depressive symptomatology. Emotion. 2008;8(3): 379-385.
2. Hambleton RK. The next generation of the ITC test translation and adaptation guidelines. Eur J Psychol Assess. 2001;17(3): 164-172.
3. Olatunji BO, Williams NL, Tolin DF, Abramowitz JS, Sawchuk CN, Lohr JM, Elwood LS. The Disgust Scale: item analysis, factor structure, and suggestions for refinement. Psychol Assess. 2007;19(3): 281-97.
4. Beck AT, Steer RA, Brown GK. Manual for the Beck Depression Inventory-II. San Antonio, TX: Psychological Corporation; 1996.
5. Fountoulakis KN, Iacovides A, Kleanthous S, Samolis S, Gougoulias K, Kaprinis, ST, et al. The Greek translation of the symptoms rating scale for depression and anxiety: preliminary results of the validation study. BMC Psychiatry. 2003;3(1): 21-28.
6. Robins RW, Hendin HM, Trzesniewski KH. Measuring global self-esteem: Construct validation of a single-item measure and the Rosenberg Self-Esteem Scale. Pers Soc Psychol Bull. 2001;27(2): 151-161.
7. Koumi I, Tsiantis J. Smoking trends in adolescence: Report on a Greek school-based peer-led intervention aimed at prevention. Health Promot Int. 2001;16(1): 65-72.
8. Marsh HW, Barnes J, Cairns L, Tidman M. Self-Description Questionnaire: Age and sex effects in the structure and level of self-concept for preadolescent children. J Educ Psychol. 1984;76(5): 940-956.
9. Tsorbatzoudis H. Psychometric evaluation of the Greek physical self-description questionnaire. Percept Mot Skills. 2005;101(1): 79-89.
10. Simpson J, Hillman R, Crawford T, Overton PG. Self-esteem and self-disgust both mediate the relationship between dysfunctional cognitions and depressive symptoms. Motiv Emot. 2010;34(4): 399-406.
11. Arbuckle J, Wothke W. AMOS 4 user’s reference guide. Chicago: Small waters Corporation; 1999.
12. Thompson B, Daniel LG. Factor analytic evidence for the construct validity of scores: A historical overview and some guidelines. Educ Psychol Meas. 1996;56(2): 197-208.
13. Hu L, Bentler PM. Cutoff criteria for fit indices in covariance structure analysis: Conventional criteria versus new alternatives. Struct Equ Modeling. 1999;6(1): 1–55.
14. Powell PA, Overton PG, Simpson J. The revolting self: Perspectives on the psychological, social, and clinical implications of self-directed disgust. London: Karnac books; 2014.
15. Mesquita B, Karasawa M. Self-conscious emotions as dynamic cultural processes. Psychol Inq. 2004;15(2): 161-166.
16. Tracy JL, Robins RW, Tangney JP. The self-conscious emotions: Theory and research. Guilford Press; 2007.
17. Brycz H, Różycka-Tran J, Szczepanik J. Cross-cultural differences in metacognitive self. Economics & Sociology. 2015;8(1): 157-164.
18. Pouliasi K, Verkuyten M. Self-evaluations, psychological well-being, and cultural context: The changing Greek society. J Cross Cult Psychol. 2011;42(5): 875-890.
19. Becker M, Vignoles VL, Owe E, Brown R, Smith PB, Easterbrook M, et al. Culture and the distinctiveness motive: Constructing identity in individualistic and collectivistic contexts. J Pers Soc Psychol. 2012;102(4): 833.

**S1A Table.** Correlations between self-report measures in the SDS-G validation study.

|  | 1.BDI | 2.RES | 3.SDS-G | 4.DS-R | 5.SDQ |
| --- | --- | --- | --- | --- | --- |
| 2. | -.630** |  |  |  |  |
| 3. | .651** | -.704** |  |  |  |
| 4. | .128* | -.090 | .013 |  |  |
| 5. | -.392** | .549** | -.507** | -.092 |  |
| Mean | 10.04 | 21.08 | 27.99 | 63.07 | 280.36 |
| SD | 7.81 | 4.93 | 10.70 | 16.12 | 49.69 |

BDI= Beck Depression Inventory II; RES=Rosenberg's Self-Esteem Scale; SDS-G=Self-Disgust Scale Greek version; DS-R= Disgust Scale-Revised; SDQ= Self-Description Questionnaire; *p<.05; **p<.001.

**S1B Table.** Component Loadings in the Greek SDS (2 factors).

| **Item no.** | **Content of item** | **Component 1** | **Component 2** |
| --- | --- | --- | --- |
| 1. | I find myself repulsive | .67 |  |
| 3. | The way I behave makes me despise myself |  | .78 |
| 4. | I hate being me | .77 |  |
| 5. | I like the way I look | .62 |  |
| 7. | I feel good about the way I behave |  | .61 |
| 8. | I do not want to be seen | .60 |  |
| 9. | I often do things I find revolting |  | .48 |
| 10. | It bothers me to look at myself | .67 |  |
| 11. | I detest aspects of my personality |  | .47 |
| 12. | My behavior repels people |  | .68 |
